# Tauroursodeoxycholic acid alleviates pulmonary endoplasmic reticulum stress and epithelial-mesenchymal transition in bleomycin-induced lung fibrosis

**DOI:** 10.1186/s12890-021-01514-6

**Published:** 2021-05-05

**Authors:** Bin Tong, Lin Fu, Biao Hu, Zhi-Cheng Zhang, Zhu-Xia Tan, Se-Ruo Li, Yuan-Hua Chen, Cheng Zhang, Hua Wang, De-Xiang Xu, Hui Zhao

**Affiliations:** 1 Second Affiliated Hospital, Anhui Medical University, Hefei, 230032 China; 2 Department of Toxicology, Anhui Medical University, Hefei, 230032 China; 3 Tong Ling People’s Hospital, Tongling, 244000 China

**Keywords:** Idiopathic pulmonary fibrosis, Tauroursodeoxycholic acid, Epithelial-mesenchymal transition, Endoplasmic reticulum stress, Oxidative stress

## Abstract

**Background:**

Several studies demonstrate that endoplasmic reticulum (ER) stress-mediated epithelial-mesenchymal transition (EMT) is involved in the process of bleomycin (BLM)-induced pulmonary fibrosis. Tauroursodeoxycholic acid (TUDCA), a bile acid with chaperone properties, is an inhibitor of ER stress. This study aimed to investigate the preventive effects of TUDCA on BLM-induced EMT and lung fibrosis.

**Methods:**

The model of lung fibrosis was established by intratracheal injection with a single dose of BLM (3.0 mg/kg). In TUDCA + BLM group, mice were intraperitoneally injected with TUDCA (250 mg/kg) daily.

**Results:**

BLM-induced alveolar septal destruction and inflammatory cell infiltration were alleviated by TUDCA. BLM-induced interstitial collagen deposition, as determined by Sirius Red staining, was attenuated by TUDCA. BLM-induced elevation of pulmonary α-smooth muscle actin (α-SMA) and reduction of pulmonary E-cadherin were attenuated by TUDCA. BLM-induced pulmonary Smad2/3 phosphorylation was suppressed by TUDCA. BLM-induced elevation of Ki67 and PCNA was inhibited by TUDCA in mice lungs. In addition, BLM-induced elevation of HO-1 (heme oxygenase-1) and 3-NT (3-nitrotyrosine) was alleviated by TUDCA. Finally, BLM-induced upregulation of pulmonary GRP78 and CHOP was attenuated by TUDCA.

**Conclusions:**

These results provide evidence that TUDCA pretreatment inhibits Smad2/3-medited EMT and subsequent lung fibrosis partially through suppressing BLM-induced ER stress and oxidative stress.

**Supplementary Information:**

The online version contains supplementary material available at 10.1186/s12890-021-01514-6.

## Background

Idiopathic pulmonary fibrosis (IPF) is characterized by excessive deposition of collagen, leading to death due to the lack of effective therapies [1, 2]. Bleomycin (BLM), an efficacious anti-cancer chemotherapeutic agent, causes a dose-dependent interstitial lung fibrosis [3, 4]. The model of BLM-evoked lung fibrosis has been used extensively in animal experiments over the past years for resembling human interstitial pulmonary fibrosis [5, 6]. Although the mechanism has not completely been clarified, alveolar epithelial damage, interstitial inflammation and transforming growth factor (TGF)-β/Smad2/3-mediated epithelial-mesenchymal transition (EMT) play a vital role in the pathogenesis of BLM-induced lung fibrosis [7].

There is increasing evidence that alveolar epithelial damage and lung fibrosis are associated with oxidative stress [8]. Clinical observation found that lipid peroxide levels were higher in patients with IPF than those in healthy subjects [9]. Animal experiment showed that administration with antioxidant alleviated BLM-induced lung fibrosis [10]. On the other hand, several studies have confirmed that endoplasmic reticulum (ER) stress and unfolded protein response (UPR) are involved in BLM-induced pulmonary EMT and lung fibrosis [11, 12]. Tauroursodeoxycholic acid (TUDCA), a hydrophilic bile acid, has been effectively used to treat cholestasis [13]. Recently, several studies found that TUDCA alleviated non-liver diseases, such as intestinal inflammation and neurodegenerative disorders, through suppressing ER stress [14, 15]. However, further study is required to determine whether TUDCA alleviates BLM-induced pulmonary fibrosis.

The objective of this study is to investigate whether TUDCA has a therapeutic effect on BLM-induced EMT and subsequent lung fibrosis in a mouse model. Our results provide experimental evidence that TUDCA attenuates pulmonary EMT and subsequent lung fibrosis partially through suppressing BLM-induced oxidative stress and ER stress.

## Methods

### Animals and treatments

BLM was purchased from HiSUN PFIZER Pharmaceuticals Co., Ltd; Zhejiang, China. TUDCA was prepared from EMD Millipore Corporation; Billerica, MA. Adult male C57BL/6 J (7 weeks-old, 21–23 g) mice were provided by Beijing Vital River Laboratory Animal Technology Co., Ltd (Beijing, China) and housed under a natural day/night cycle room with comfortable environment (temperature 20–25 °C, humidity 45–50%). Mice were supplied with enough food and water. Based on the previous studies of our laboratory and power calculation analysis, 80 mice were used in this study [11, 12]. Eighty mice were divided into 4 groups randomly. The experimental protocol was shown in Additional file 1: Figure S1. In TUDCA alone and BLM + TUDCA group, mice were intraperitoneally injected with TUDCA (250 mg/kg) once a day for 21 days. In BLM alone and BLM + TUDCA group, mice were intratracheally injected with a single dose of BLM (3.0 mg/kg, 1 mg bleomycin = 1000 IU bleomycin). Mice were intraperitoneally injected with saline and administered with saline by intratracheal injection in control group. The dose of BLM and the number of mice based on existing literature [11]. The dose of TUDCA based on existing literature [16]. Two mice were died in the BLM group. All mice were euthanized at 21 d after BLM injection. Left lungs were fixed for hematoxylin and eosin (H&E) staining and Masson trichrome staining. Right lungs were harvested and homogenized in liquid nitrogen for immunoblots, immunohistochemistry and RT-PCR [17]. This study was approved by the Association of Laboratory Animal Sciences and the Center for Laboratory Animal Sciences at Anhui Medical University (Permit Number: 13–0016). All procedures on animals followed the Guide for the Care and Use of Laboratory Animals published by the US National Institutes of Health (NIH Publication No. 85–23, revised 1996).

### Histology and pulmonary collagen identification

The left lungs of the mice were collected and fixed with 4% paraformaldehyde solution. Lung tissue sections were stained with hematoxylin eosin (H&E) and observed under a light microscope by a blinded and experienced investigator. Morphological changes were scored absent (0), mild (1), moderate (2) or severe injury (3) based on the presence of exudates, hyperemia/congestion, neutrophilic infiltrates, intraalveolar hemorrhage/debris, and cellular hyperplasia. HE staining and pathological scores were conducted based on the existing literature [11]. Mice in BLM-exposed group showed dim hair, loss of appetite and decreased activity. However, the health condition was better in control, TUDCA and BLM + TUDCA groups than these in BLM group. Collagen deposition in lung tissue was measured through Sirius Red staining. The percentage of collagen deposition area was determined using Image J Pro software. The quantification of images was performed by two investigators which were blinded to the experimental groups. Ten mice per group were used. Twelve images were randomly selected from similar lung lobe in every mouse at ×200 magnification. The hydroxyproline assay were performed in mice lungs according to the previous study [18].

### Immunoblots

The protein extraction and quantitative analysis were carried out based on the existing literature [19]. Briefly, mouse lung tissue was fully lysed in RIPA buffer and the appropriate protein concentration was determined. For immunoblots, equal amounts of protein from different treatment groups were added in sodium lauryl sulfate polyacrylamide gel and transferred to the membranes when the protein ladder was electrophoresed to the appropriate position. The membranes were incubated with different primary antibodies (α-SMA, phosphorylated-Smad2, phosphorylated-Smad3, Smad2/3, etc.) for different time. The membranes were washed in the PBST solution for three times, followed by incubating with different secondary antibodies. Antibodies against Ki-67, GRP78, E-cadherin, CHOP (C/EBP-homologous protein) and p-Smad2 were provided by Cell Signaling Technology (Beverley, MA). Antibodies against PCNA, α-SMA, p-Smad3 and Smad2/3 were provided by Abcam (Cambridge, UK). Antibody against β-actin was provided by Sigma Chemical Co., (St. Louis, MO). Antibodies against HO-1, 3-NT and secondary antibodies were provided by Santa Cruz Biotechnology Inc. (Santa Cruz, CA). Antibodies information was shown in Supplemental Table 1. Membrane signal was detected using ECL detection kit (Advansta Inc., California, USA). β-actin was used as a reference in the present study. Ten mice per group were used. Representative blots were selected and used in the figures.

### Immunohistochemistry (IHC)

Pulmonary α-SMA, p-Smad3, 3-NT and Ki67 were detected using immunohistochemistry (IHC). Briefly, lung tissues were fixed in paraformaldehyde, dehydrated and embedded. Endogenous peroxidases were blocked in 3% H_2_O_2_. Antigen retrieval was performed in the boiled citrate buffer for 4 min at an interval of 5 min, total 3 times. After inactivation of endogenous peroxidase and serum blocking, primary antibodies were incubated at 4℃ overnight. Then, goat anti-rabbit and goat anti-mouse IgG (1: 1000) were incubated 2 h at 37℃. Eventually, aspirate the primary antibody with a pipette, color reaction was detected with biotin-streptavidin peroxidase system [20]. Moreover, in order to suppress nonspecific staining, positive and negative controls were performed in this study. Negative control was defined as which primary antibody or second antibody were replaced with PBS. Positive control was defined as which lung sections of confirmed pulmonary fibrosis in the previous study were performed through IHC staining [11, 12]. Ten mice per group were used. Representative images were selected and used in the figures.

### RNA extraction and RT-PCR analysis

Total RNA extraction was referred to existing literature [21] and cDNAs were produced by reverse transcription reagent kit (Promega, Madison, USA). Real-time RT-PCR was conducted in LightCycler480 Instrument (Roche Diagnostics, Germany). Primers for real-time RT-PCR were listed as following: *Tgf-β* (F, TTCCGCTGCTACTGCAAGTCA; R, GGGTAGCGATCGAGTGTCCA), *18S* (F, GTAACCCGTTGAACCCCATT; R, CCATCCAATCGGTAGTAGCG). Gene expression was normalized to *18S*.

### Statistical analyses

Statistical analyses were performed using SPSS software (version 23.0). Figures were produced using GraphPad Prism 7.0. The normality of distribution was analyzed using Kolmogorov–Smirnov test. Continuous variables were expressed as means ± S.E.M. or medians with interquartile ranges. Differences among different groups in normally distributed data were analyzed by ANOVA and Tukey's test. Data non-normally distributed were analyzed by nonparametric test (Kruskal–Wallis test and Mann–Whitney U test). *P* < 0.05 was regarded as statistically significant.

## Results

### TUDCA mitigates BLM-induced pulmonary histological damage and collagen deposition in mice

**Fig. 1:**
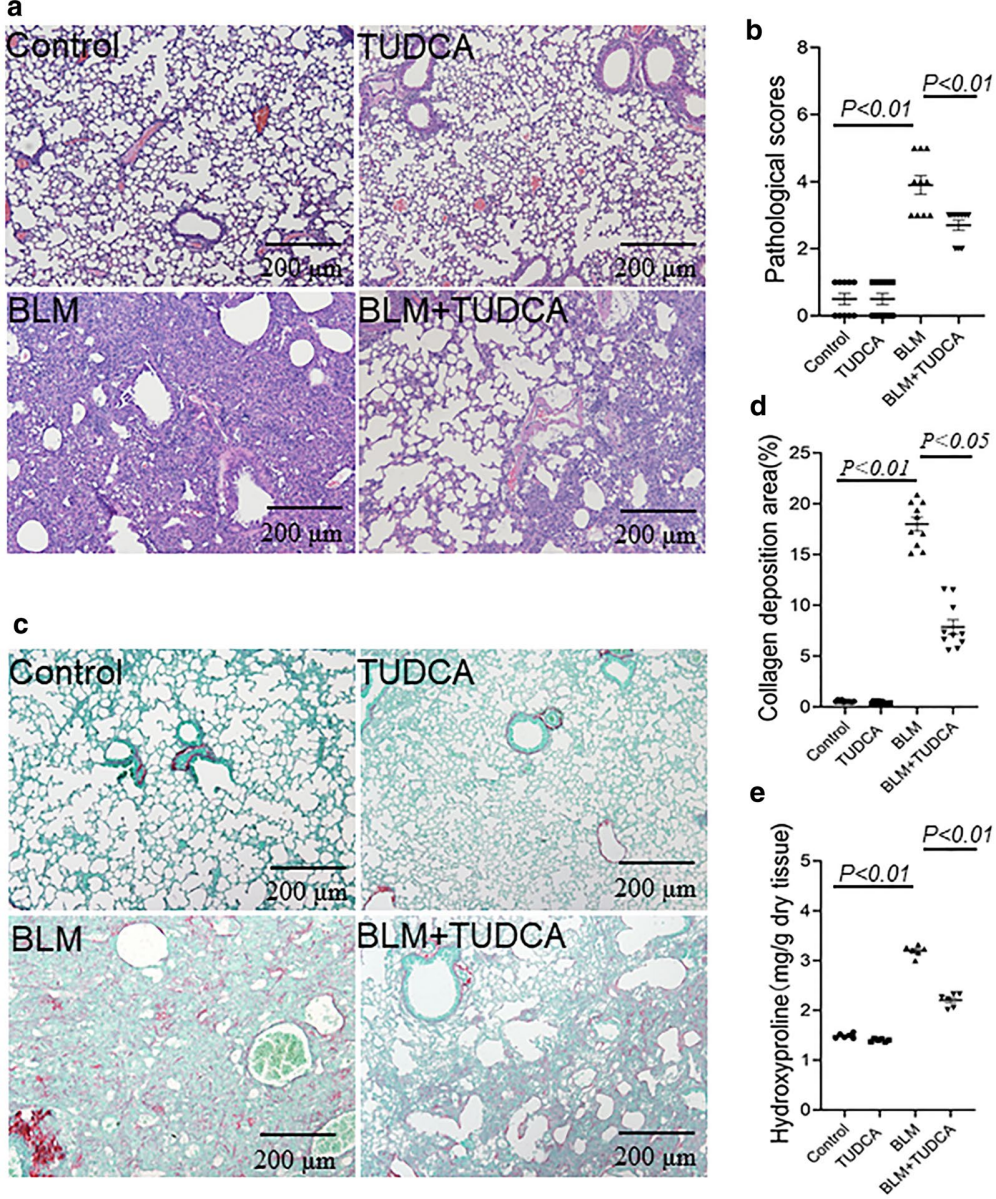
TUDCA attenuates BLM-induced histological damage and collagen deposition in the mice lungs. In BLM alone and BLM + TUDCA group, mice were intratracheally injected with a single dose of BLM (3.0 mg/kg). In TUDCA alone and BLM + TUDCA group, mice were intraperitoneally injected with TUDCA (250 mg/kg) once a day. Mice were intraperitoneally injected with NS and administered with NS by intratracheal injection in control group. Mice were sacrificed at 21 d after BLM treatment. **a** Pulmonary tissues were stained with H&E. Original magnification: 100×. **b** Pathological damage degree was assessed. **c** Pulmonary tissues were stained with Sirius Red. Original magnification: 100×. **d** Quantification of collagen deposition area. **e** The levels of hydroxyproline were detected in mice lungs. All data were expressed as means ± S.E.M. (pathological damage degree and levels of hydroxyproline) or median (IQR) (collagen deposition area) of ten lung tissues from ten different mice

There was no death in Control and TUDCA groups. Bleomycin exposure induced death on the 4–7th and 14–17th days after the administration. Eight mice were died in the process of BLM-induced pulmonary fibrosis. There was no difference of healthy condition between Control and TUDCA groups. BLM exposure induced a dim hair, decreased appetite, activity and body weight. The healthy condition was better in Control and TUDCA groups than these in BLM and BLM + TUDCA groups. The average weight in four groups was as follows: Control: 25.0 g, TUDCA: 24.3 g, BLM: 19.5 g, BLM + TUDCA: 21.1 g. To investigate whether TUDCA alleviate BLM-induced pathological damage and collagen deposition of pulmonary fibrosis. H&E staining and Sirius Red staining was performed on lung tissue sections. According to H&E staining, the main histological damages in BLM-treated mice was alveolar septal destruction. Interestingly, pretreatment with TUDCA attenuated BLM-induced alveolar septal destruction in mice lungs (Fig. 1a, b). Sirius Red staining showed a large amount of collagen deposition in pulmonary interstitium of BLM-treated mice. Pretreatment with TUDCA markedly attenuated BLM-induced collagen deposition in pulmonary interstitium (Fig. 1c, d). Moreover, the levels of hydroxyproline were detected in mice lungs. The results found that BLM-exposure elevated the levels of hydroxyproline in mice lungs (Fig. 1f). Interestingly, pretreatment with TUDCA alleviated BLM-induced up-regulation of hydroxyproline (Fig. 1f).

### TUDCA inhibits BLM-induced pulmonary EMT in mice

**Fig. 2:**
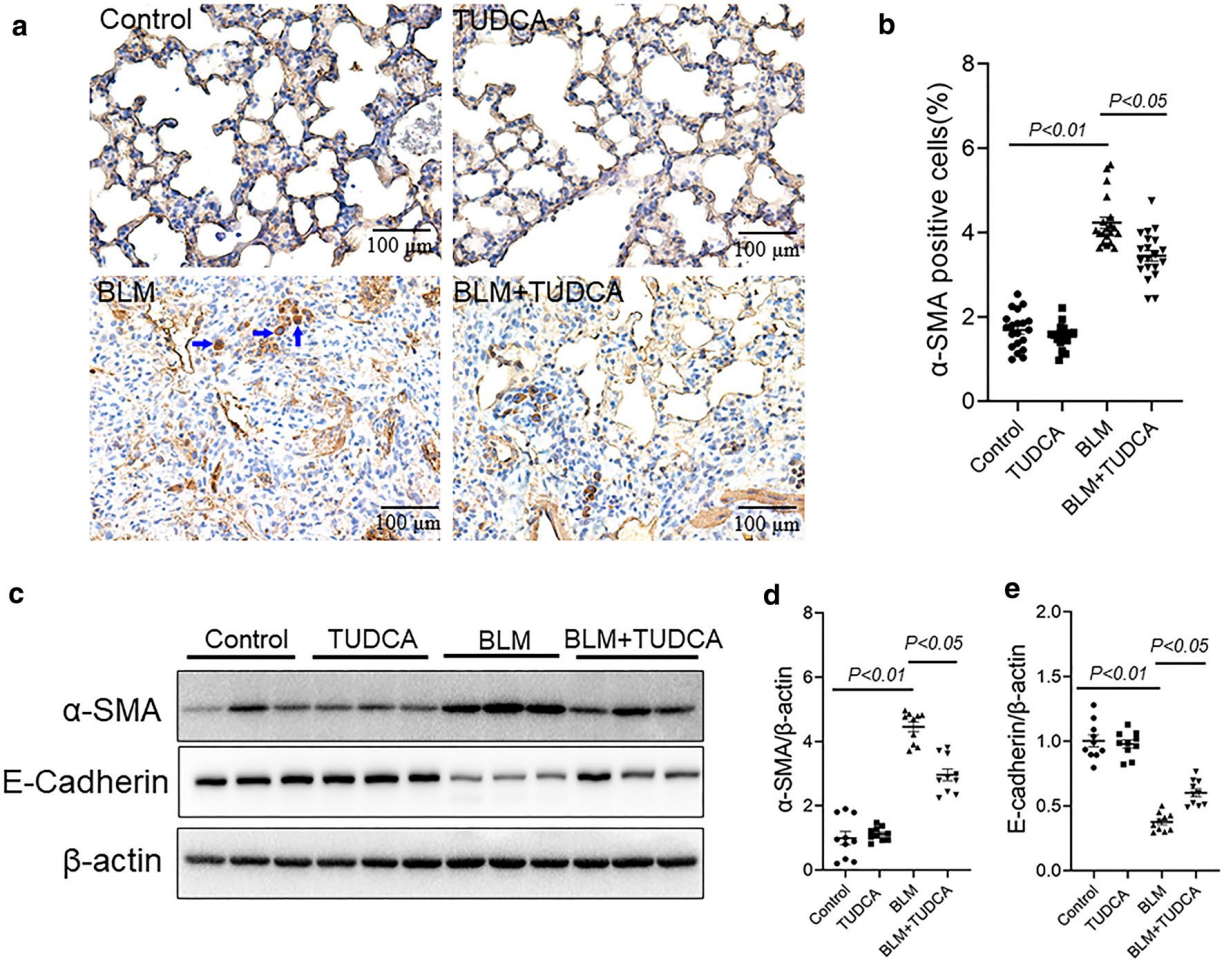
TUDCA inhibits BLM-induced EMT in the mice lungs. In BLM alone and BLM + TUDCA group, mice were intratracheally injected with a single dose of BLM (3.0 mg/kg). In TUDCA alone and BLM + TUDCA group, mice were intraperitoneally injected with TUDCA (250 mg/kg) once a day. Mice were intraperitoneally injected with NS and administered with NS by intratracheal injection in control group. Mice were sacrificed at 21 d after BLM treatment. **a** Pulmonary α-SMA-positive cells were detected via IHC. Original magnification: 400×. **b** Pulmonary α-SMA-positive cells were counted. **c** α-SMA and E-cadherin were detected via immunoblot. **d**, **e** Quantitative analysis of grayscale value. All data were expressed as means ± S.E.M. of ten lung tissues from ten different mice

EMT plays a crucial role in pulmonary fibrosis [12]. α-SMA is a hallmark of myofibroblasts and is generally accepted as a marker for EMT. E-cadherin, an epithelial marker, is an important cell adhesion molecule. To explore whether TUDCA alleviate pulmonary EMT in BLM-treated mouse lungs, α-SMA and E-cadherin were measured. The results indicated that the percentage of pulmonary α-SMA-positive cells was markedly elevated in the BLM-treated mice. After pretreatment with TUDCA, the percentage of pulmonary α-SMA-positive cells was reduced (Fig. 2a, b). Further analysis displayed that the expression of pulmonary α-SMA was elevated by BLM. Correspondingly, pretreatment with TUDCA downregulated α-SMA in the lungs of BLM-treated mice (Fig. 2c, d). By contrast, the expression of pulmonary E-cadherin was obviously decreased in BLM-treated mice lungs. After pretreatment with TUDCA, BLM-induced downregulation of pulmonary E-cadherin was inhibited (Fig. 2c, e).

### TUDCA alleviates BLM-induced pulmonary TGF-β/Smad2/3 activation in mice

**Fig. 3:**
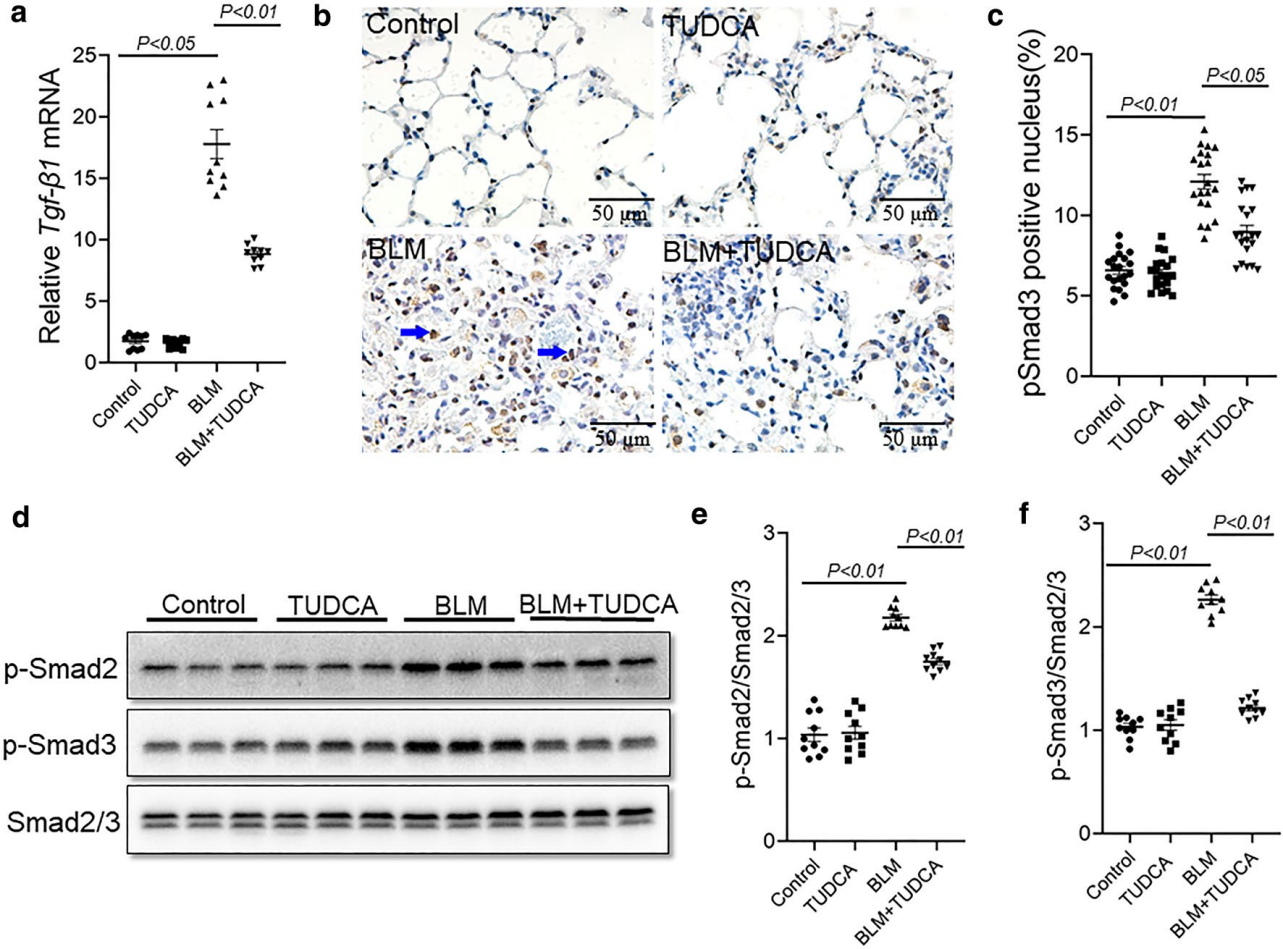
TUDCA alleviates BLM-induced activation of TGF-β/Smad2/3 in the mice lungs. In BLM alone and BLM + TUDCA group, mice were intratracheally injected with a single dose of BLM (3.0 mg/kg). In TUDCA alone and BLM + TUDCA group, mice were intraperitoneally injected with TUDCA (250 mg/kg) once a day. Mice were intraperitoneally injected with NS and administered with NS by intratracheal injection in control group. Mice were sacrificed at 21 d after BLM treatment. **a** Pulmonary *Tgf-β1* mRNA were detected by real-time RT-PCR. **b** Pulmonary p-Smad3-positive cells were detected by IHC. Original magnification: 400×. **c** Pulmonary p-Smad3-positive cells were counted. **d** Pulmonary p-Smad2 and p-Smad3 were detected via immunoblot. **e**, **f** Quantitative analysis of grayscale value. All data were expressed as means ± S.E.M. (p-Smad2 and p-Smad3) or median (IQR) (*Tgf-β1* mRNA and p-Smad3-positive cells) of ten lung tissues from ten different mice

Smad2/3-mediated EMT of alveolar epithelial cells is involved in the pathogenesis of BLM-induced lung fibrosis [22, 23]. To investigate whether TUDCA alleviate BLM-induced pulmonary TGF-β/Smad2/3 activation in mice, TGF-β/Smad2/3 signaling was detected. Pulmonary *Tgf-β1* mRNA level was upregulated after BLM treatment in mice lungs. After pretreatment with TUDCA, BLM-induced upregulation of pulmonary *Tgf-β1* mRNA was inhibited (Fig. 3a). As shown in IHC, pulmonary p-Smad3-positive cells were elevated by BLM treatment. After pretreatment with TUDCA, BLM-induced elevation of p-Smad3-positive cells was significantly attenuated (Fig. 3b, c). In addition, the levels of pulmonary p-Smad2 and p-Smad3 were elevated after BLM treatment. After pretreatment with TUDCA, BLM-induced phosphorylation of pulmonary Smad2 and Smad3 was inhibited (Fig. 3d–f).

### TUDCA inhibits BLM-induced cell proliferation in mice lungs

**Fig. 4:**
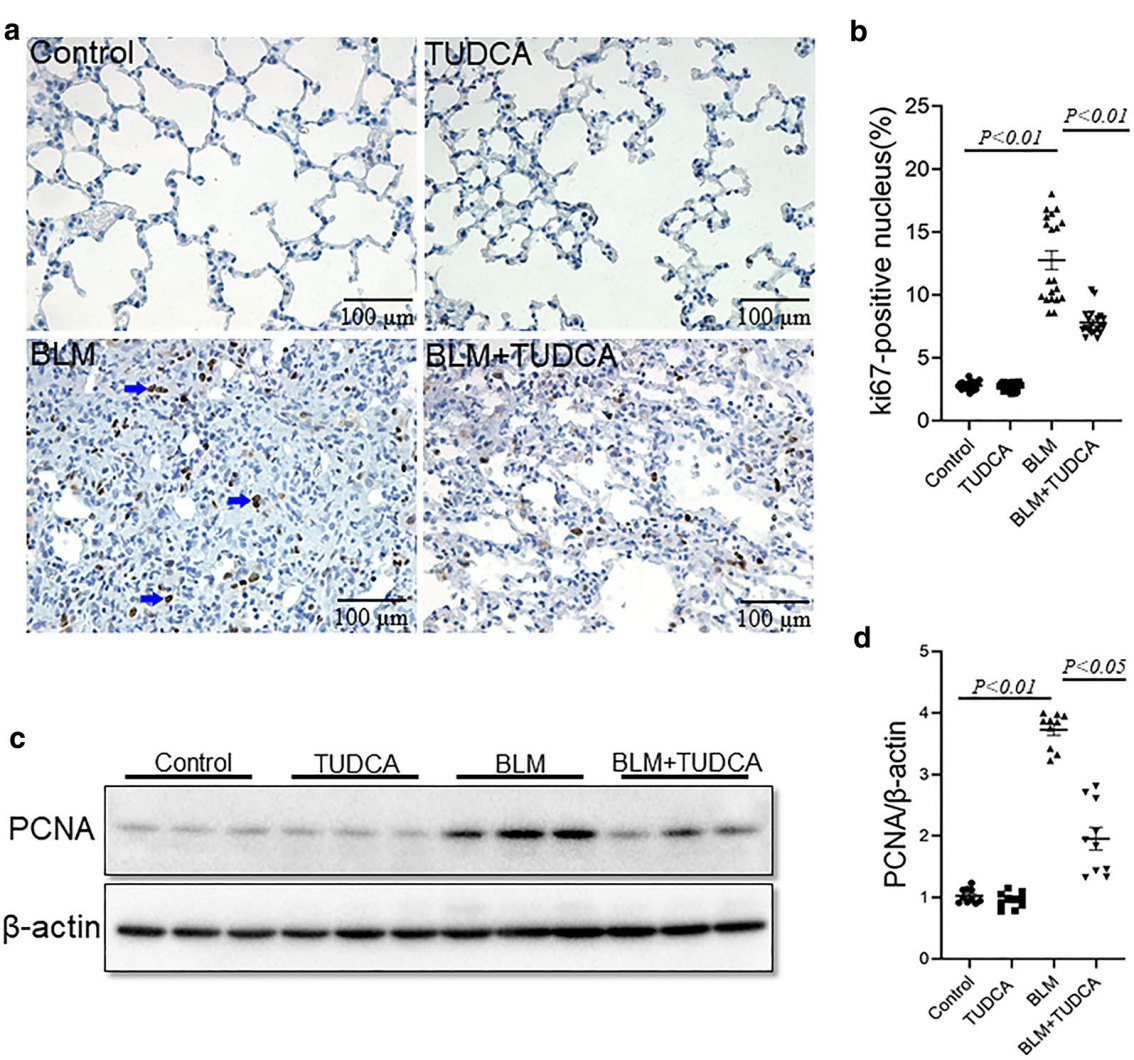
TUDCA inhibits BLM-induced cell proliferation in mouse lungs. In the BLM alone and BLM + TUDCA groups, mice were intratracheally injected with a single dose of BLM (3.0 mg/kg). In the TUDCA alone and BLM + TUDCA groups, mice were intraperitoneally injected with TUDCA (250 mg/kg) once a day. Mice were sacrificed at 21 d after BLM treatment. **a** Pulmonary Ki67-positive cells was detected by IHC. Original magnification: 400×. **b** Pulmonary Ki67-positive cells were counted. **c** Pulmonary PCNA was detected by immunoblot. **d** Quantitative analysis of grayscale value. All data were expressed as median (IQR) of ten lung tissues from ten different mice

In the present study, two markers of cellular proliferation were detected. Ki67 was detected by IHC. PCNA was detected by immunoblot. As shown in Fig. 4a, b, pulmonary Ki67-positive cells, were increased in BLM-treated mice. Of interest, BLM-induced cellular proliferation was alleviated in TUDCA-pretreated mice (Fig. 4a, b). The effect of TUDCA on pulmonary PCNA protein was explored. As shown in Fig. 4c, d. As expected, pulmonary PCNA level was increased in BLM-treated mice. TUDCA pretreatment obviously attenuated BLM-induced elevation of PCNA in mice lungs (Fig. 4c, d).

### TUDCA attenuates BLM-induced upregulation of HO-1 and 3-NT in mice lungs

**Fig. 5:**
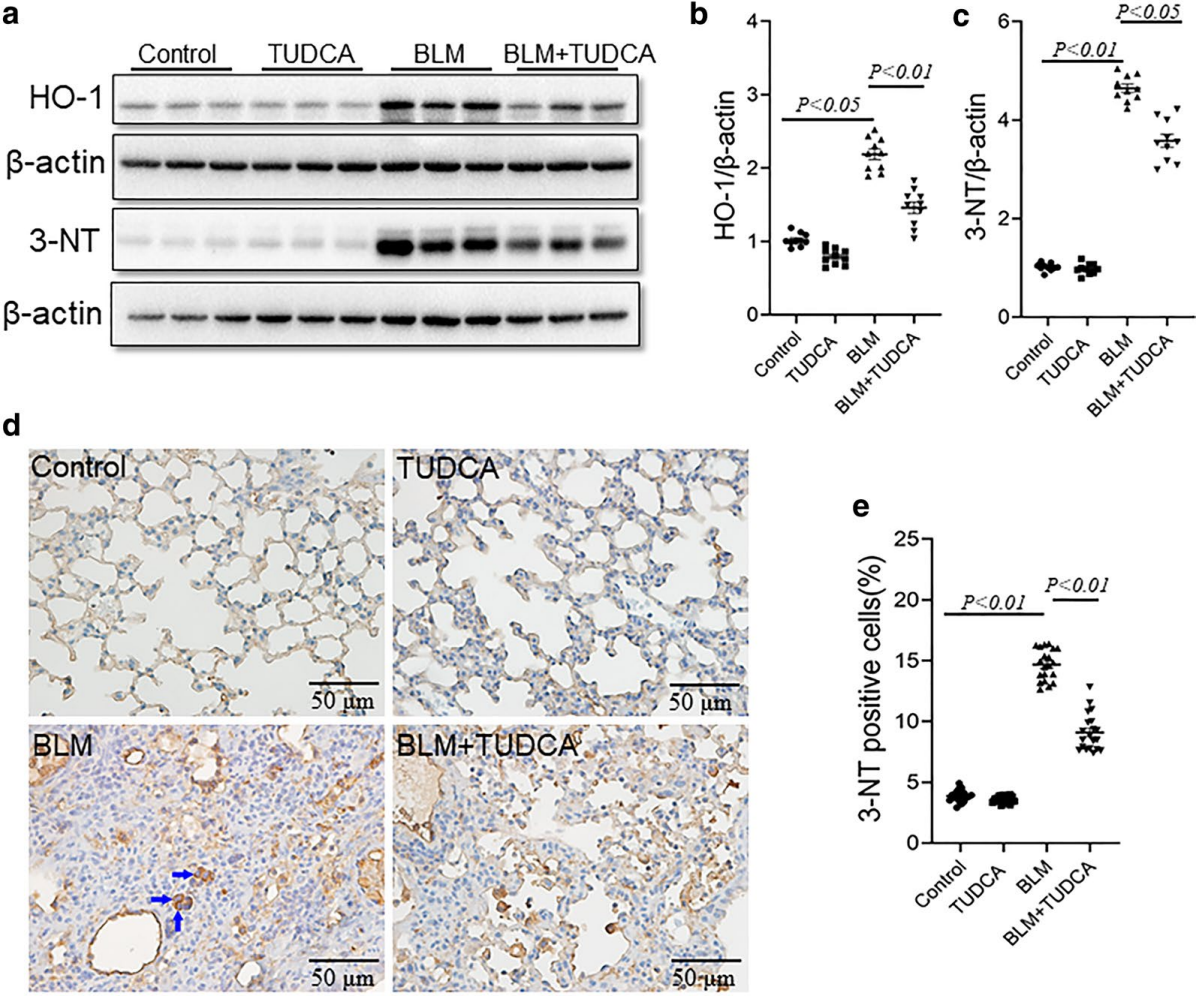
TUDCA attenuates BLM-induced oxidative stress in the lungs. In BLM alone and BLM + TUDCA group, mice were intratracheally injected with a single dose of BLM (3.0 mg/kg). In TUDCA alone and BLM + TUDCA group, mice were intraperitoneally injected with TUDCA (250 mg/kg) once a day. Mice were intraperitoneally injected with NS and administered with NS by intratracheal injection in control group. Mice were sacrificed at 21 d after BLM treatment. **a** Pulmonary HO-1 and 3-NT were detected by immunoblot. **b**, **c** Quantitative analysis of grayscale value. **d** Pulmonary 3-NT-positive cells was detected by IHC. Original magnification: 400×. **e** Pulmonary 3-NT-positive cells were counted. All data were expressed as median (IQR) of ten lung tissues from ten different mice

Increasing data have demonstrated that excess ROS (reactive oxygen species) takes part in the process of BLM-evoked lung fibrosis [24, 25]. HO-1 and 3-NT, two markers of oxidative stress, were detected in mice lungs. In the present study, we found that pulmonary HO-1 protein was increased after BLM treatment. After pretreatment with TUDCA, BLM-induced increase of HO-1 was inhibited in mice lungs (Fig. 5a, b). 3-NT was elevated in BLM-treated mice. After pretreatment with TUDCA, pulmonary 3-NT was alleviated (Fig. 5a, c). IHC showed that 3-NT-positive cells were increased in the lungs of mice after BLM treatment. After pretreatment with TUDCA, the percentage of pulmonary 3-NT-positive cells was alleviated (Fig. 5d, e).

### TUDCA relieves BLM-induced pulmonary ER stress in mice

**Fig. 6:**
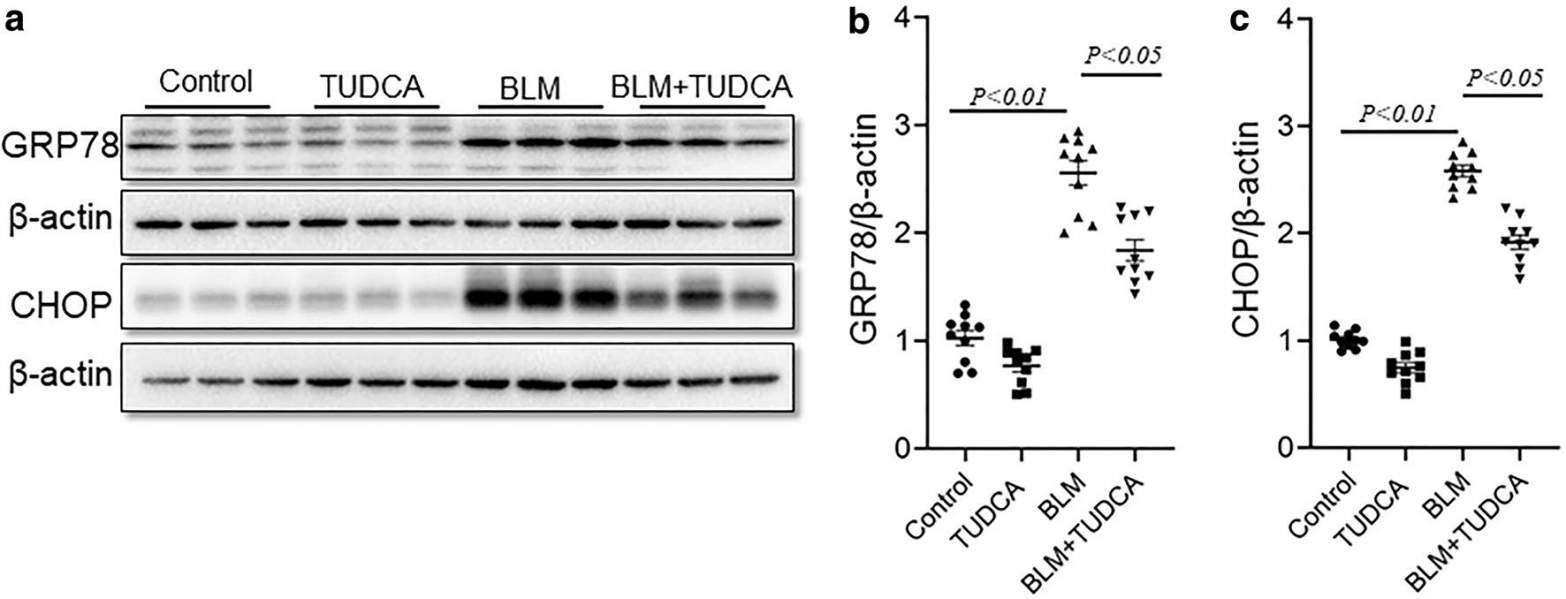
TUDCA blocks BLM-induced ER stress in the lungs. In BLM alone and BLM + TUDCA group, mice were intratracheally injected with a single dose of BLM (3.0 mg/kg). In TUDCA alone and BLM + TUDCA group, mice were intraperitoneally injected with TUDCA (250 mg/kg) once a day. Mice were intraperitoneally injected with NS and administered with NS by intratracheal injection in control group. Mice were sacrificed at 21 d after BLM treatment. **a** Pulmonary GRP78 and CHOP were detected via immunoblot. **b**, **c** Quantitative analysis of grayscale value. All data were expressed as means ± S.E.M. (CHOP) or median (IQR) (GRP78) of ten lung tissues from ten different mice

GRP78, an ER chaperone and a marker of ER stress, was elevated in lungs of BLM-treated mice. After pretreatment with TUDCA, the expression of GRP78 was decreased (Fig. 6a, b). In addition, CHOP, a marker of UPR, was increased in BLM-treated mouse lungs. After pretreatment with TUDCA, BLM-evoked elevation of CHOP was suppressed (Fig. 6a, c).

## Discussion

In this study, we evaluated whether TUDCA pretreatment had a prophylactic effect on BLM-induced lung fibrosis in mice. Our results suggested that TUDCA alleviated BLM-induced pulmonary EMT and subsequent lung fibrosis. The specific findings include: (1) TUDCA pretreatment inhibits BLM-induced pulmonary TGF-β/Smad2/3 signaling activation; (2) TUDCA pretreatment prevents the elevation of α-SMA and reversed the reduction of E-cadherin during BLM-induced lung fibrosis; (3) TUDCA alleviates BLM-evoked collagen deposition in the mice lungs.

Several studies suggest that pulmonary ER stress plays an important role in BLM-induced lung fibrosis [11, 12]. TUDCA, a chemical molecular chaperone, has been widely used to improve ER function and protein-folding homeostasis [26]. Several studies found that TUDCA attenuated hepatic and cardiac fibrosis by inhibiting ER stress [27, 28]. According to a recent report, TUDCA alleviated acute kidney injury and renal fibrosis through suppressing ischemia/reperfusion-induced ER stress [16]. In the current study, the effect of TUDCA on pulmonary ER stress was explored during BLM-induced lung fibrosis. As expected, pulmonary GRP78, a marker of ER stress, was upregulated. CHOP, a marker of the UPR, was ascended in the lungs of BLM-treated mice. TUDCA inhibited upregulation of pulmonary GRP78 and CHOP in the lungs of BLM-treated mice. These results suggest that TUDCA pretreatment prevents pulmonary fibrosis, at least partially, through suppressing BLM-evoked ER stress in mice lungs.

Smad2/3-mediated EMT of alveolar epithelial cells is involved in the pathogenesis of BLM-induced lung fibrosis [22, 23]. In this study, we found that pulmonary TGF-β1 was upregulated and Smad2/3 was activated in BLM-exposed mice lungs. Moreover, the number of α-SMA-positive cells, a marker of pulmonary EMT, was elevated after BLM treatment. The level of pulmonary α-SMA protein was also upregulated after BLM intratracheal instillation. By contrast, pulmonary E-cadherin, an epithelial marker, was downregulated during BLM-induced lung fibrosis. Two early reports indicated that EMT is accompanied by ER stress in alveolar epithelial cells [29, 30]. The current study explored the effect of TUDCA on TGF-β/Smad2/3-mediated EMT in BLM-induced lung fibrosis. Our results showed that TUDCA alleviated BLM-induced TGF-β1 upregulation and Smad2/3 activation in the lungs. Moreover, TUDCA inhibited α-SMA upregulation and E-cadherin downregulation in BLM-evoked lung fibrosis. These results provide evidence that TUDCA pretreatment prevents BLM-induced lung fibrosis through inhibiting EMT in the lungs.

Though, the pathogenesis of IPF remains unclear. Nowadays, it is thought that IPF always results from an abnormal wound healing response to epithelial injury in genetically susceptible individuals [31]. Moreover, the present study found that anti-inflammatory and immunosuppressive agents cannot treat this disease effectively, meaning that chronic inflammatory may be not only cause of IPF [32]. Increasing data suggest that excessive proliferation of fibroblasts is involved in the development of IPF [33, 34]. An early study found that BLM promoted the proliferation of fibroblasts [35]. Ki67 and PCNA are two markers of cellular proliferation [36, 37]. A recent study found that TUDCA inhibited proliferation of fibroblasts during lung fibrosis [38]. In the present study, we showed that pulmonary Ki67 positive cells were increased in BLM-treated mice. Moreover, pulmonary PCNA protein was upregulated in BLM-treated mice. Of interest, TUDCA inhibited BLM-induced elevation of Ki67-positive cells in mice lungs. Moreover, TUDCA attenuated BLM-induced upregulation of PCNA in mice lungs. These results provide additional evidence that TUDCA pretreatment prevents BLM-induced pulmonary fibrosis partially through inhibiting cellular proliferation in lungs.

Increasing data have demonstrated that excess ROS taken part in the process of BLM-evoked lung fibrosis [24, 25]. N-acetylcysteine, an antioxidant, can effectively protect against BLM-induced lung fibrosis [39–41]. In this study, our results showed that the levels of HO-1 and 3-NT, two markers of oxidative stress, were increased after BLM treatment. The number of 3-NT-positive cells was elevated in BLM-induced lung fibrosis. Indeed, TUDCA has an antioxidant activity [42–44]. The present study found that TUDCA alleviated BLM-induced elevation of pulmonary HO-1 and 3-NT. Therefore, the present study does not exclude that TUDCA pretreatment protects against lung fibrosis through suppressing BLM-induced oxidative stress.

In this study, we have focused on protection effect of TUDCA pretreatment against TGF-β/Smad2/3-mediated EMT in the process of BLM-induced lung fibrosis. Nevertheless, there are a few limitations in this study. Firstly, the current study only investigated the preventive effect on BLM-induced pulmonary EMT and subsequent lung fibrosis by using a single dose of TUDCA. This is a prophylactic experiment. However, the treatment effect of TUDCA on BLM-induced lung fibrosis is not unclear. Secondly, this study did not explore the exact mechanism which TUDCA inhibited TGF-β/Smad2/3-mediated EMT in BLM-evoked lung fibrosis. Thirdly, only BLM-induced pulmonary fibrosis model was used in the current study. Because it has been found to have the possibility of self-recovery in a mouse model and species difference, multiple administrations and new suitable fibrosis models are needed in the future work. Thus, further research is necessary to investigate the effects of different TUDCA doses on BLM-evoked EMT and lung fibrosis. Fourthly, only part markers of EMT were measured in mice lungs. In order to evaluate the effect of TUDCA on BLM-evoked EMT, more in vitro experiments should be performed. Different pulmonary epithelial cells were selected and used. The change of morphology in pulmonary epithelial cells was observed. Additionally, wound healing, migration and invasion should be conducted in pulmonary epithelial cells. Besides, the levels of mRNAs and protein of markers in EMT, included E-cadherin, ZO-1, N-cadherin, Vimentin, α-SMA and Fibronectin, should be measured using RT-PCR and western blotting. Moreover, E-cadherin and α-SMA were also detected through immunohistochemistry. Not only that, EMT related nuclear transcription factors, such as Snail, ZEB and Twist, would be evaluated using western blotting and immunofluorescence.

## Conclusion

In summary, the aim of this study is to explore the effects of TUDCA on BLM-induced lung fibrosis in a mouse model. Our results showed that TUDCA suppressed pulmonary TGF-β/Smad2/3-mediated EMT in the process of BLM-induced lung fibrosis. We found that TUDCA alleviated pulmonary cell proliferation and collagen deposition during BLM-induced lung fibrosis. The present study provides experimental evidence that TUDCA prevents pulmonary EMT and subsequent lung fibrosis partially through suppressing BLM-induced oxidative stress and ER stress. Therefore, TUDCA may be used as a potential prophylactic drug for lung fibrosis.

## Supplementary Information

**Additional file 1**. The experimental protocol.

**Additional file 2**. Antibodies information.

## Data Availability

Prof. Hui Zhao should be contacted if someone wants to request the data from this study.

## Abbreviations

IPF: Idiopathic pulmonary fibrosis
BLM: Bleomycin
TUDCA: Tauroursodeoxycholic acid
EMT: Epithelial-mesenchymal transition
ER: Endoplasmic reticulum
HO-1: Heme oxygenase-1
3-NT: 3-nitrotyrosine
α-SMA: α-Smooth muscle actin
TGF: Transforming growth factor
UPR: Unfolded protein response
ROS: Reactive oxygen species
CHOP: C/EBP-homologous protein
