# Supplementary figures and images for: Tauroursodeoxycholic acid alleviates pulmonary endoplasmic reticulum stress and epithelial-mesenchymal transition in bleomycin-induced lung fibrosis
Source: BMC Pulm Med. 2021 May 5;21:149. doi: 10.1186/s12890-021-01514-6 (PMC8097922; doi:10.1186/s12890-021-01514-6)

**Supplementary Data**


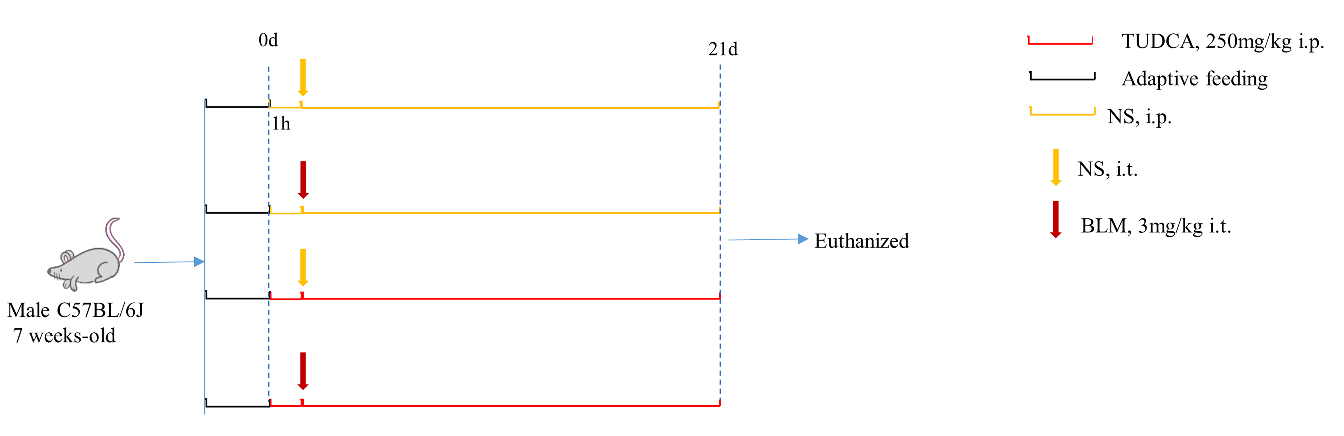


**Supplemental Figure 1.** The experimental protocol.

Supplement: Supplementary file 1 — Additional file 1. The experimental protocol. [file 12890_2021_1514_MOESM1_ESM.docx]
