# Supplementary material for: Tauroursodeoxycholic acid alleviates pulmonary endoplasmic reticulum stress and epithelial-mesenchymal transition in bleomycin-induced lung fibrosis
Source: BMC Pulm Med. 2021 May 5;21:149. doi: 10.1186/s12890-021-01514-6 (PMC8097922; doi:10.1186/s12890-021-01514-6)
Supplement: Supplementary file 2 — Additional file 2. Antibodies information. [file 12890_2021_1514_MOESM2_ESM.docx]

Supplemental Table 1. Antibodies information.

| Antibodies | Company | Applications | Primary antibody | Secondary antibody |
| --- | --- | --- | --- | --- |
| Alpha-SMA (ab7817) | Abcam | WB | 1:2000 | 1:60000 |
|  |  | IHC | 1:200 | 1:1000 |
| Smad3 (phospho S423+S425, ab52903) | Abcam | WB | 1:2000 | 1:60000 |
|  |  | IHC | 1:400 | 1:1000 |
| Smad2/3 (ab217553) | Abcam | WB | 1:2000 | 1:60000 |
| phospho-Smad2 (Ser465/Ser467, #18338) | CST | WB | 1:2000 | 1:60000 |
| BiP (CST, #3177) | CST | WB | 1:2000 | 1:60000 |
| CHOP (#2895) | CST | WB | 1:2000 | 1:60000 |
| Ki-67 (#9449) | CST | IHC | 1:200 | 1:1000 |
| E-Cadherin (#14472) | CST | WB | 1:2000 | 1:60000 |
| PCNA (sc-56) | Santa Cruz | WB | 1:2000 | 1:60000 |
| HO-1 (sc-390991) | Santa Cruz | WB | 1:2000 | 1:60000 |
| 3-NT (sc-32757) | Santa Cruz | WB | 1:2000 | 1:60000 |
|  |  | IHC | 1:200 | 1:1000 |
